# Optimization of callus culture for enhanced rutaecarpine and evodiamine accumulation in *Tetradium daniellii*

**DOI:** 10.3389/fpls.2026.1827737

**Published:** 2026-05-13

**Authors:** Ji Won Kim, Chae-Bin Lee, Sun-Cheon Hong, Sung-Joon Na, Ji-Min Park, Kyu-Suk Kang

**Affiliations:** 1 Department of Agriculture, Forestry, and Bioresources, College of Agriculture and Life Sciences, Seoul National University, Seoul, Republic of Korea; 2 Department of Forest and Conservation Sciences, Faculty of Forestry, University of British Columbia, Vancouver, BC, Canada; 3 Special Forest Resources Division, National Institute of Forest Science, Suwon, Republic of Korea; 4 Research Institute of Agriculture and Life Sciences, Seoul National University, Seoul, Republic of Korea

**Keywords:** callus culture, evodiamine, rutaecarpine, secondary metabolites, *Tetradium daniellii*, UHPLC-MS/MS

## Abstract

*Tetradium daniellii* (Benn.) T.G. Hartley, native to Korea and China, is valued as a melliferous tree traditionally used as a medicinal plant. The species contains alkaloids such as evodiamine and rutaecarpine, with evodiamine exhibiting anticancer activity and rutaecarpine showing anti-inflammatory, cardioprotective, and anti-obesity bioactivities. However, these compounds are mainly extracted from fruits, and their content varies depending on harvest time and region, while repeated harvesting may lead to resource depletion. Therefore, this study aimed to identify optimal conditions for enhancing production of these alkaloids and to compare their levels across tissues and culture conditions to assess feasibility of tissue culture-based production systems. After 8 weeks of *in vitro* growth from sterilized seeds, leaves and petioles of plantlets were cultured on MS, WPM, DKW, and SH media. The cultures were incubated under two conditions: either a light (16 h light/8 h dark) condition or continuous dark conditions (24 h dark) to induce callus formation. The induced callus, immature fruits, and plantlets were analyzed using Ultra High-Performance Liquid Chromatography-Mass Spectrometry (UHPLC-MS/MS). Callus induction reached 100% in WPM medium for both leaf and petiole tissues regardless of light conditions. Petiole tissues also exhibited high induction rates under light condition on MS medium, while DKW medium showed high re-differentiation rates exceeding 70%. In contrast, SH medium showed low induction rates or limited callus proliferation. Quantitative analysis revealed that rutaecarpine content was highest in callus cultured on MS medium, reaching 140.83 mg·kg^-1^, approximately 217 times higher than *in vitro*-grown leaves and 5.7 times higher than immature fruit. Evodiamine was also most abundant under the same conditions, reaching 6.45 mg·kg^-1^, representing a 72-fold increase compared with leaf tissues and a 1.2-fold increase relative to immature fruit. These results demonstrate that callus cultures showed higher accumulation of both alkaloids than conventional tissues and fruits. Leaf-derived callus cultured on MS medium under light conditions was the most effective treatment for enhancing alkaloid accumulation. These findings provide a useful basis for understanding the factors influencing alkaloid accumulation in callus cultures and offer insights for the development of *in vitro* systems, contributing to the sustainable utilization of plant resources.

## 1 Introduction

*Tetradium daniellii* (Benn.) T.G. Hartley is a deciduous tree species belonging to the Rutaceae family within the order Sapindales. It is native to Korea, including Ganghwa Island, Gyeonggi Province, and Ulleungdo Island, as well as certain regions of China (Ri, 1997). Blooming from July to August when few other nectar-producing plants are in flower, it provides abundant nectar resources (Ryu et al., 2007) and is widely utilized in ecological restoration and as an important nectar resource in apiculture (Choi et al., 2019).

Beyond its ecological and industrial significance, members of the Rutaceae family are well known as natural sources of bioactive indole alkaloids, particularly evodiamine and rutaecarpine. These compounds have been studied in the fruit of *Evodia rutaecarpa* (Chen and Chen, 1933), where they exhibit notable anticancer (Jiang and Hu, 2009; Son et al., 2015; Liu et al., 2022), anti-inflammatory (Liu et al., 2009), and cardioprotective activities (Hu et al., 2002), thereby establishing fruit tissue as the primary source for their extraction. However, fruit-based production is inherently constrained by environmental variability and limited availability (Yang et al., 2018; Yeshi et al., 2022; Zhan et al., 2022), restricting the stable and consistent supply of these metabolites. These limitations highlight the need to explore alternative plant materials and culture systems for sustainable alkaloid production in related species including *T*. *daniellii*.

Plant tissue culture offers a controlled and sustainable alternative for producing secondary metabolites independent of environmental fluctuations (Reshi et al., 2023; Prashant and Bhawana, 2024). Among these approaches, callus culture enables rapid biomass proliferation (Murthy et al., 2014), thereby providing a suitable platform for investigating and enhancing biosynthetic pathways. Despite the recognized pharmacological potential of *T*. *daniellii*, studies on callus induction and the regulation of alkaloid biosynthesis remain scarce; no optimized culture system has yet been established to enhance evodiamine or rutaecarpine accumulation.

This study aims to identify culture conditions that promote callus induction and influence alkaloid accumulation in *T*. *daniellii*. By comparing medium compositions, light conditions, and tissue types, including callus derived under different culture conditions, immature fruit, and *in vitro* grown leaf tissues, it provides the first systematic evaluation of factors associated with dedifferentiation and indole alkaloid accumulation in this species. These findings offer initial insights into the regulation of alkaloid accumulation in *T*. *daniellii* and establish a foundation for future studies on *in vitro* culture systems.

## 2 Materials and methods

### 2.1 Plant materials

Seeds of *Tetradium daniellii* were collected from Wonju, Gangwon-do, Republic of Korea in October 2023. Mature tissues of *T*. *daniellii* were collected from the experiment forest of special tree species, operated by the National Institute of Forest Science, located at San 21, Wonpyeong-ri, Maesong-myeon, Hwaseong-si, Gyeonggi-do, Republic of Korea. Immature fruits were harvested on September 23, 2024.

To achieve *in vitro*-grown plantlets, seeds were surface sterilized with 70% ethanol for 30 seconds and 2% sodium hypochlorite solution for 10 minutes, and then rinsed five times with sterile distilled water. After sterilization, seeds were cultured on Murashige and Skoog medium (MS) supplemented with 1.0 mg·L^-1^ gibberellic acid (GA_3_), 0.5 mg·L^-1^ 6-Benzylaminopurine (6-BAP), 3% (w/v) sucrose, and 0.3% (w/v) gelrite. The medium pH was adjusted to 5.8 prior to autoclaving. Cultures were maintained at 25 °C and 70% relative humidity under photoperiod of 16 h light/8h dark and continuous darkness for 8 weeks. The light intensity in the culture room was 6-8 μmol m^-2^ s^-1^ photosynthetic photon flux density (PPFD), as measured using an LI-250A light meter (LI-COR, USA).

### 2.2 Callus induction

Leaf and petiole tissues were excised from *in vitro*-grown plantlets maintained for 8 weeks. The explants were cut into 2–4 mm segments and cultured on MS (Murashige and Skoog, 1962), Woody Plant Medium (WPM) (Lloyd and McCown, 1980), Driver and Kuniyuki Walnut (DKW) (Driver and Kuniyuki, 1984), and Schenk and Hildebrandt (SH) (Schenk and Hildebrandt, 1972) media supplemented with 1.0 mg·L^-1^ 2,4-Dichlorophenoxyacetic acid (2,4-D) and 0.5 mg·L^-1^ BAP, 3% (w/v) sucrose, and 0.3% (w/v) gelrite, maintained at 5.8 pH. Cultures were incubated at 25 ± 2 °C under either light (16 h light/8 h dark) or continuous dark (24 h) conditions for 6 weeks.

**Figure 1:**
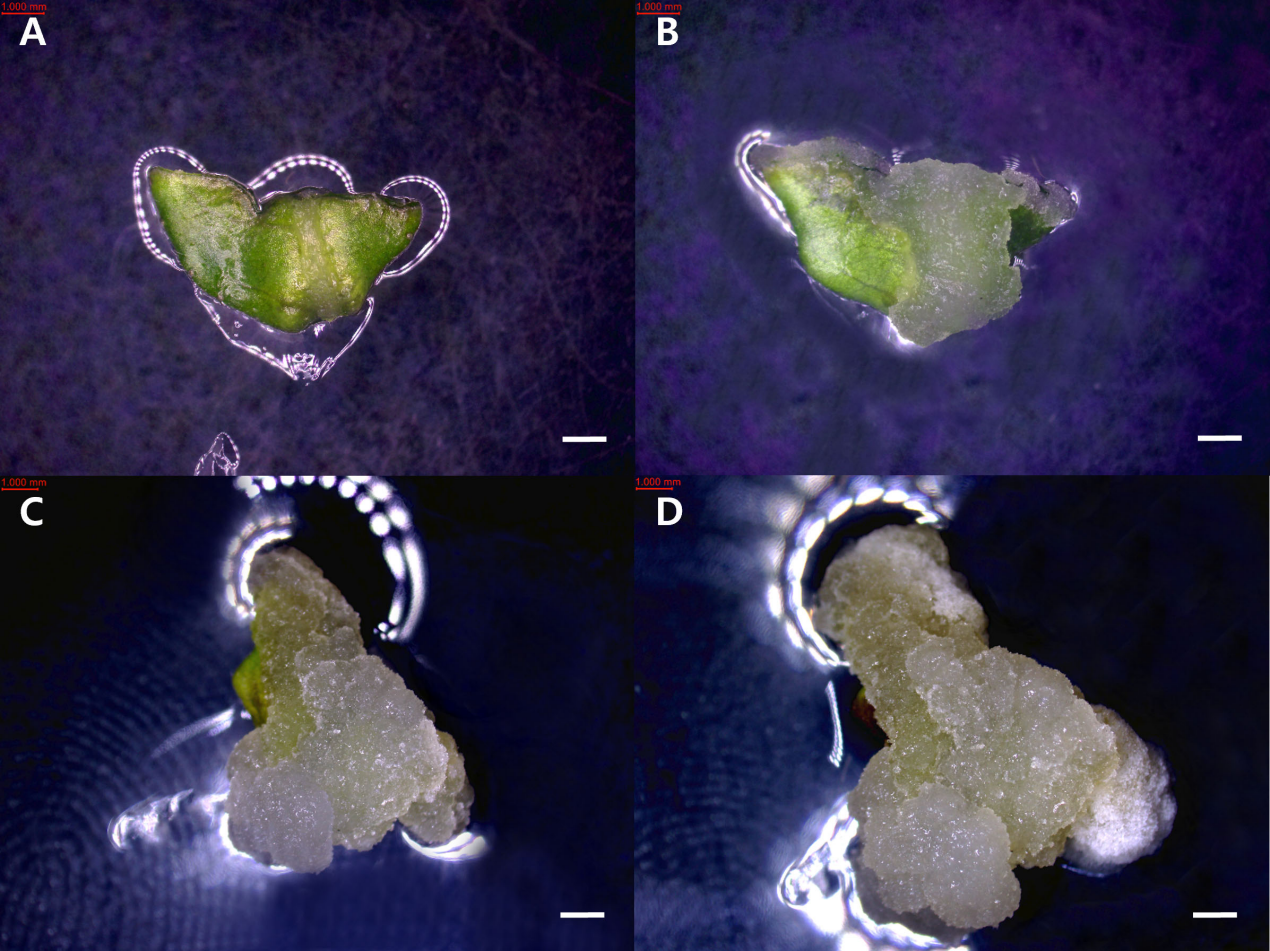
Developmental stages of callus induction from *Tetradium daniellii* leaf explants cultured *in vitro*. **(A)** Stage 1, **(B)** Stage 2, **(C)** Stage 3, and **(D)** Stage 4. Scale bar represents 1 mm.

**Figure 2:**
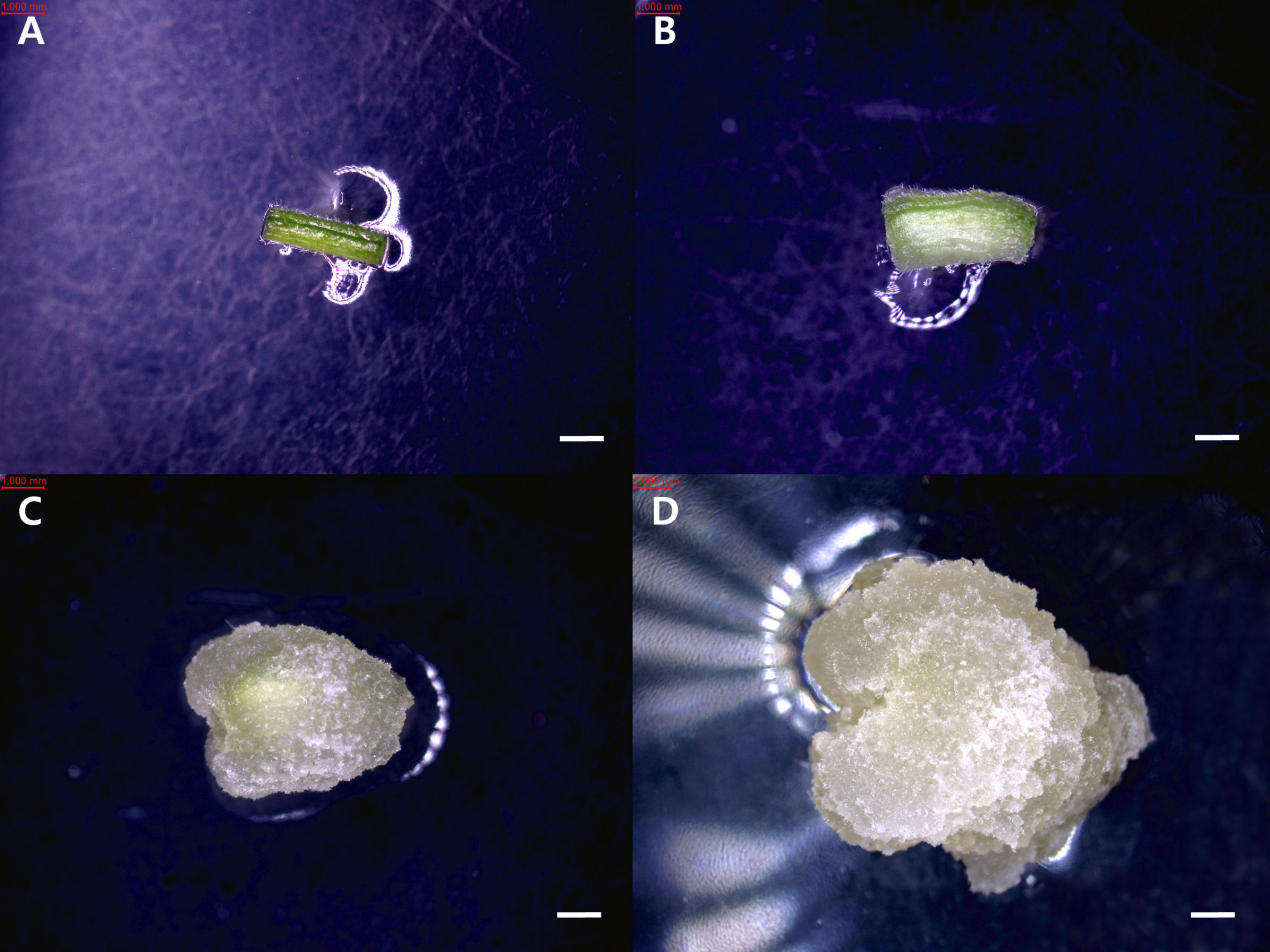
Developmental stages of callus induction from *T. daniellii* petiole explants cultured *in vitro*. **(A)** Stage 1, **(B)** Stage 2, **(C)** Stage 3, and **(D)** Stage 4. Scale bar represents 1 mm.

Callus induction rates were calculated as the number of explants forming callus divided by the total number of cultured explants, multiplied by 100. The redifferentiation rate was calculated as the number of callus that produced organs divided by the total number of cultured explants, multiplied by 100. Callus developmental stages were observed at two-week intervals under a stereomicroscope (Nikon, SMZ745T) and classified into four categories based on morphological characteristics. In Stage 1, no visible callus was formed, and the explant maintained its original tissue structure. Stage 2 was characterized by localized swelling and initial appearance of callus tissue on the surface of the explant. In Stage 3, the callus expanded to cover more than 50% of the cut surface. Finally, Stage 4 represented extensive callus proliferation, in which the entire explant surface was covered, and the original tissue morphology was no longer distinguishable (**Figures 1**, **2**).

All treatments were conducted using three biological replicates, each consisting of 10 independently cultured explants under identical conditions.

### 2.3 Sample preparation and extraction

Callus samples were selected from treatments that produced sufficient biomass across all replicates. Samples were freeze-dried at −110 °C for 24 hours using a HyperCOOL Cooling Trap HC3110 (GYROZEN Co., Korea). Immature fruits and leaf tissues from *in vitro*-grown plantlets were subsequently dried for an additional 16 hours (total drying time, 40 h). Dried samples were pulverized using a tissuelyser II (Qiagen, Germany), and approximately 20.0 mg of powdered material was accurately weighed using a microbalance. In a small number of samples, 16.3–18.8 mg was used due to limited biomass. The quantified concentrations obtained from UHPLC-MS/MS analysis (ng·mL^-1^) were converted to mg·kg^-1^ based on the extraction volume and the actual dry weight of each sample.

Each sample was extracted with 1.0 mL of 80% methanol (methanol:water = 8:2, v/v) by ultrasonic-assisted extraction for 60 minutes at room temperature using a Power Sonic 420 ultrasonic cleaner (Hwashin Technology Co., Korea). The extracts were centrifuged at 15,000 rpm for 10 min at 4 °C, and 800 µL of the supernatant was collected for UHPLC-MS/MS analysis.

For quantitative analysis, each treatment was analyzed using three biological replicates, and each replicate was independently extracted and prepared for subsequent UHPLC-MS/MS analysis.

### 2.4 UHPLC-MS/MS analysis

Quantitative analysis of evodiamine and rutaecarpine was performed using a Thermo Vanquish Ultra High-Performance Liquid Chromatography (UHPLC) system (Thermo Scientific, USA) coupled to a TSQ Altis triple quadrupole mass spectrometer (Thermo Fisher Scientific, USA). Separation was achieved on a Cortecs C18 column (2.1 × 50 mm, 1.6 μm; Waters, USA) maintained at 45 °C. The mobile phases consisted of water containing 0.1% formic acid (A) and acetonitrile (B), delivered at a flow rate of 0.3 mL·min^-1^. The gradient elution program was as follows: 0–1 min, 5% B; 1–3 min, linear gradient to 95% B; 3–4 min, 95% B isocratic; 4–4.1 min, decrease to 5% B; 4.1–5 min, re-equilibration at 5% B. The injection volume was 1.0 µL.

Mass spectrometric detection was performed in positive ion mode using a heated electrospray ionization (H-ESI) source under selected reaction monitoring (SRM) mode. The following source parameters were applied: spray voltage, 3,500 V; capillary temperature, 350 °C; gas flow, 10 L·min^-1^; nebulizer pressure, 40 psi; sheath gas temperature, 400 °C; sheath gas flow, 11 L·min^-1^; and capillary voltage, 4,000 V.

SRM transitions and data acquisition were controlled using TraceFinder 4.1 software. For evodiamine, the precursor ion m/z 304.1 was monitored with product ions at m/z 134.042 (quantifier), 161.0, and 171.054 (qualifiers), with a retention time of 3.06 min and collision energies ranging from 18.5 to 20.5 V. For rutaecarpine, the precursor ion m/z 288.05 was monitored with product ions at m/z 244.071 (quantifier), 271.042, and 273.042 (qualifiers), with a retention time of 3.13 min and collision energies between 32.7 to 40.8 V.

### 2.5 Calibration curves

Quantitative analysis was performed in two independent analytical runs, and for each run, separate calibration curves were constructed using authentic standards of evodiamine and rutaecarpine. For *in vitro*-grown leaf tissue extracts, calibration standards ranged from 0 to 200 ng·mL^-1^ for both compounds; for all other sample types, calibration ranges were 0–500 ng·mL^-1^ for evodiamine and 0–1,000 ng·mL^-1^ for rutaecarpine.

The quantified concentrations were, in all cases, above the limit of quantification (LOQ), which was defined as the lowest calibration level meeting acceptable accuracy and precision criteria. The LOQ values were 10 ng·mL^-1^ for evodiamine and 5 ng·mL^-1^ for rutaecarpine, while for *in vitro*-grown leaf tissue extracts they were 2 ng·mL^-1^ for evodiamine and 5 ng·mL^-1^ for rutaecarpine. All calibration curves demonstrated excellent linearity, with coefficients of determination (R²) exceeding 0.9998 in both analytical runs.

**Figure 3:**
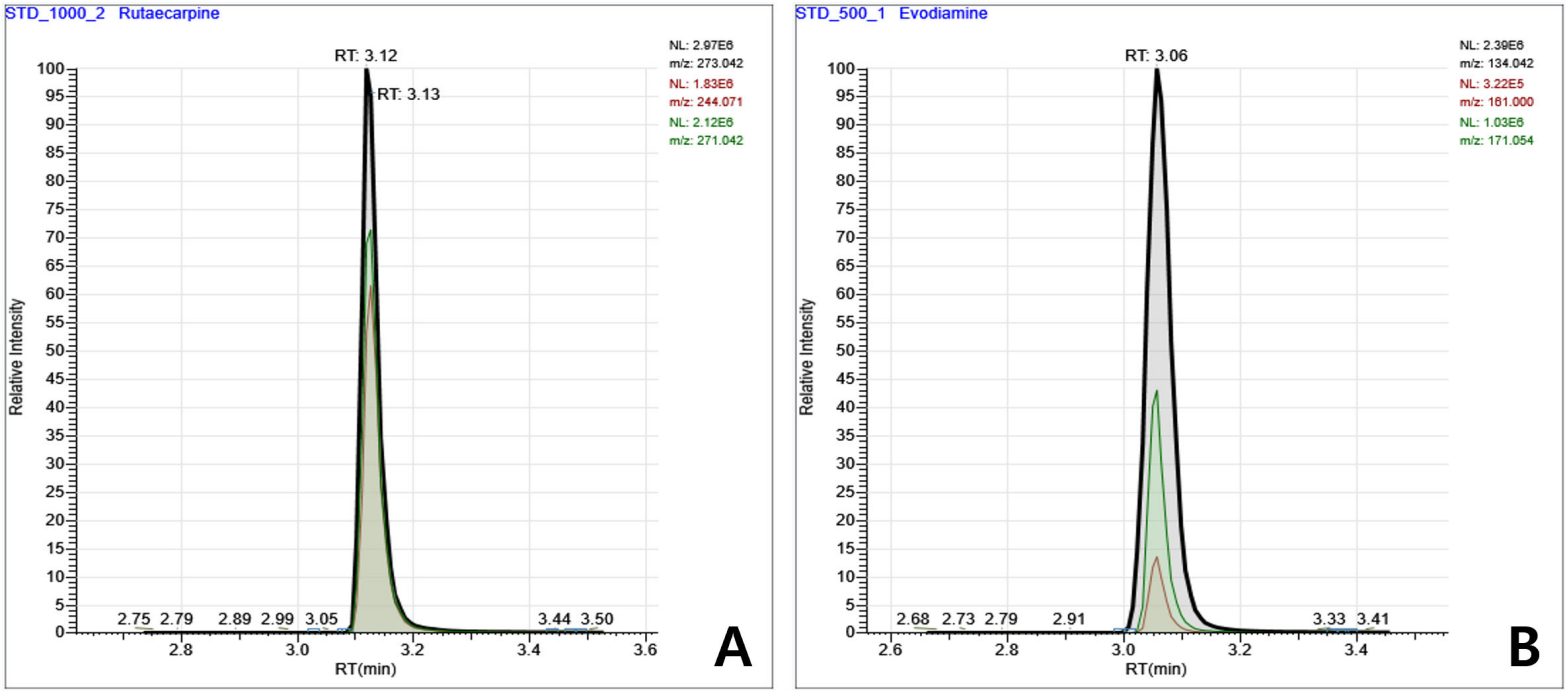
Representative selected reaction chromatograms of evodiamine and rutaecarpine standard solutions. **(A)** Evodiamine (m/z 134.042, RT = 3.06 min) and **(B)** rutaecarpine (m/z 273.042, RT = 3.12–3.13 min).

Representative extracted ion chromatograms of evodiamine and rutaecarpine standards are shown in **Figure 3**, with retention times of approximately 3.06 min and 3.12-3.13 min, respectively.

### 2.6 Statistical analysis

Statistical analyzes were conducted using IBM SPSS Statistics for Windows, Version 29.0.1.0 (IBM Corp., Armonk, NY, USA). One-way analysis of variance (ANOVA) was used to compare alkaloid contents among multiple treatment groups. Two-way ANOVA was applied to evaluate the effects of medium composition and light condition, as well as their interaction, on evodiamine and rutaecarpine accumulation. Where significant differences were detected, Duncan’s multiple range test was used for *post-hoc* comparisons at p < 0.05. All data visualizations and graphical outputs were generated using GraphPad Prism 10.1.0 (GraphPad Software, San Diego, CA, USA).

## 3 Results

### 3.1 Callus induction

#### 3.1.1 Leaf explants

Callus induction from leaf tissues of *in vitro*-grown *Tetradium daniellii* showed a significant main effect of light condition, with overall induction rates being significantly higher under light conditions than under dark conditions (F = 4.829, *p =* 0.043) (**Table 1**). In contrast, the effect of culture medium was not statistically significant, and no significant interaction between medium and light condition was detected (*p* > 0.05), indicating that light was the primary factor of callus induction from leaf tissues, independent of the culture medium. WPM medium achieved a 100% callus induction rate under both light and dark conditions - the highest efficiency among all media tested – while DKW (96.7%), MS and SH (93.3% each) also exhibited high induction rates under light conditions. Under dark conditions, however, induction rates were comparatively lower in DKW (73.3%) and SH (76.7%) media.

**Table 1:** Callus induction rate, redifferentiation rate, and developmental stage of callus derived from *in vitro* plantlet leaf tissue of *T*. *daniellii* under different media and light conditions.

Media	Light condition	Callus induction rate (%)	Redifferentiation rate (%)	Stage of callus
MS	Light	93.3 ± 6.7^*^	13.3 ± 6.7^b^	2.8
	Dark	90.0 ± 5.8	13.3 ± 8.8^b^	3.2
WPM	Light	100.0 ± 0.0^*^	50.0 ± 5.8^a^	3.2
	Dark	100.0 ± 0.0	53.3 ± 12.0^a^	3.7
DKW	Light	96.7 ± 3.3^*^	33.3 ± 17.6^ab^	3.0
	Dark	73.3 ± 14.5	26.7 ± 6.7^ab^	2.4
SH	Light	93.3 ± 6.7^*^	3.3 ± 3.3^b^	2.2
	Dark	76.7 ± 6.7	3.3 ± 3.3^b^	1.8

Values for callus induction rate and redifferentiation rate are expressed as mean ± standard error (n = 3). Callus developmental stage values represent mean scores derived from an ordinal classification system are presented without statistical grouping. Different letters indicate significant differences for callus induction rate and redifferentiation rate only according to Duncan’s multiple range test (*p* < 0.05). Asterisks (*) indicate light conditions with significant main effects on callus induction rate (p < 0.05, two-way ANOVA).

**Figure 4:**
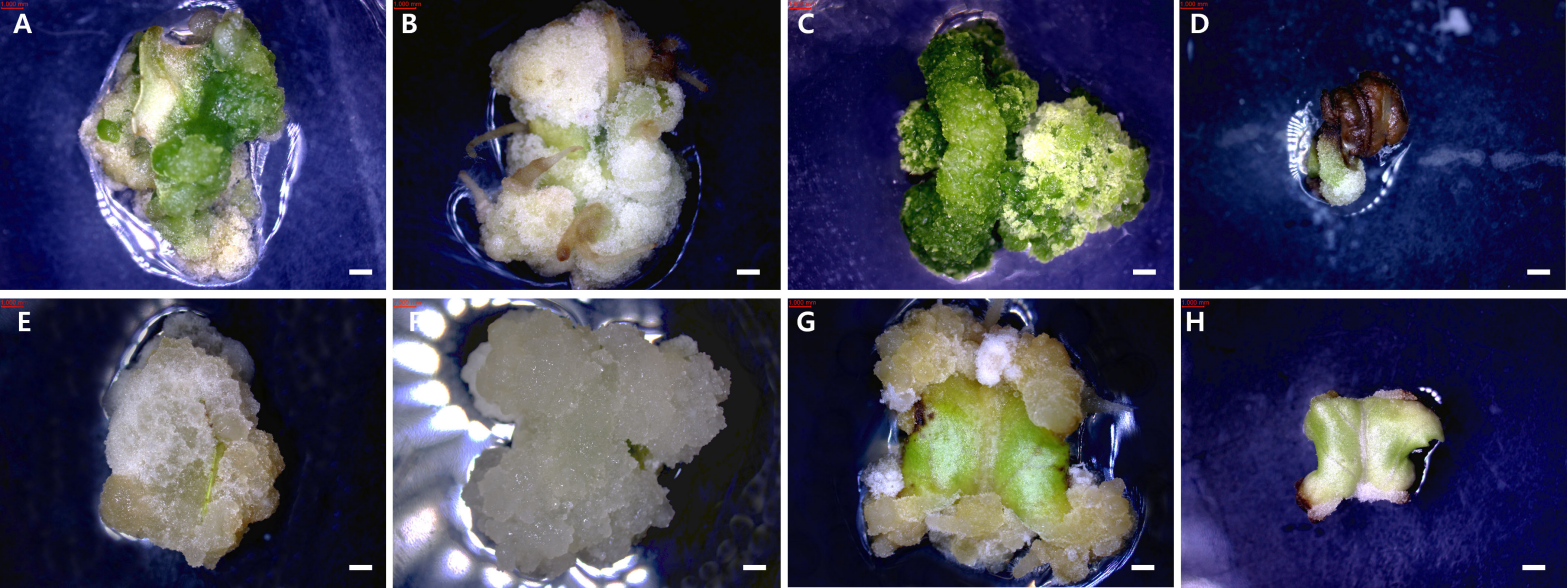
Callus development from *in vitro* plantlet leaf explants of *T*. *daniellii* after 6 weeks of culture under different media and light conditions. **(A–D)** Cultured under light conditions on MS, WPM, DKW, and SH media, respectively. **(E–H)** Cultured under dark conditions on MS, WPM, DKW, and SH media, respectively. Scale bar represents 1 mm.

The developmental stage of callus varied depending on the culture medium. Specifically, the dark condition of WPM medium showed the highest developmental level among all conditions, followed by the light condition of WPM medium (**Figure 4**). In contrast, callus cultured on SH medium under both light and dark conditions showed lower developmental scores.

Similarly, the redifferentiation rate was significantly affected only by culture medium (F = 22.065, *p* < 0.001). WPM medium showed the highest redifferentiation rates under both light (50.0%) and dark (53.3%) conditions, while relatively high rates were also observed in DKW medium under light (33.3%) and dark (26.7%) conditions. In contrast, MS medium showed a low redifferentiation rate of 13.3%, and SH medium showed 3.3%. According to Duncan’s multiple range test, WPM medium was classified into the statistically highest group, whereas SH medium belonged to the lowest group.

#### 3.1.2 Petiole explants

Callus induction rates were significantly affected by both culture medium (F = 13.525, *p* < 0.001) and light condition (F = 4.787, *p* = 0.044), whereas the interaction was not significant. A 100.0% callus induction rate was observed under both light and dark conditions in WPM medium, as well as under light conditions in MS medium (**Table 2**). A high induction rate of 93.3% was also observed under dark conditions in MS medium, confirming that it belonged to the statistically highest group according to Duncan’s multiple range test (*p* < 0.05). In contrast, the lowest induction rate was observed in SH medium under dark conditions (36.7%), which was classified into the lowest group. Overall, callus induction rates under light conditions were significantly higher than those under dark conditions.

**Table 2:** Callus induction rate, redifferentiation rate, and developmental stage of callus derived from *in vitro* plantlet petiole tissue of *T*. *daniellii* under different media and light conditions.

Media	Light condition	Callus induction rate (%)	Redifferentiation rate (%)	Stage of callus
MS	Light	100.0 ± 0.0^a*^	6.7 ± 6.7^b^	3.8
	Dark	86.7 ± 13.3^a^	3.3 ± 3.3^b^	3.4
WPM	Light	100.0 ± 0.0^a*^	60.0 ± 10.0^a^	4.0
	Dark	100.0 ± 0.0^a^	50.0 ± 17.3^a^	3.9
DKW	Light	96.7 ± 3.3^a*^	70.0 ± 20.8^a^	3.8
	Dark	96.7 ± 3.3^a^	76.7 ± 3.3^a^	3.7
SH	Light	73.3 ± 16.7^b*^	6.7 ± 3.3^b^	1.8
	Dark	36.7 ± 6.7^b^	0.0 ± 0.0^b^	1.4

Values for callus induction rate and redifferentiation rate are expressed as mean ± standard error (n = 3). Callus developmental stage values represent mean scores derived from an ordinal classification system are presented without statistical grouping. Different letters represent significant differences for callus induction rate and redifferentiation rate only according to Duncan’s multiple range test (*p* < 0.05). Asterisks (*) indicate light conditions with significant main effects on callus induction rate (*p* < 0.05, two-way ANOVA). Callus induction rate was significantly affected by both medium and light, while redifferentiation rate was influenced only by medium (*p* < 0.001).

**Figure 5:**
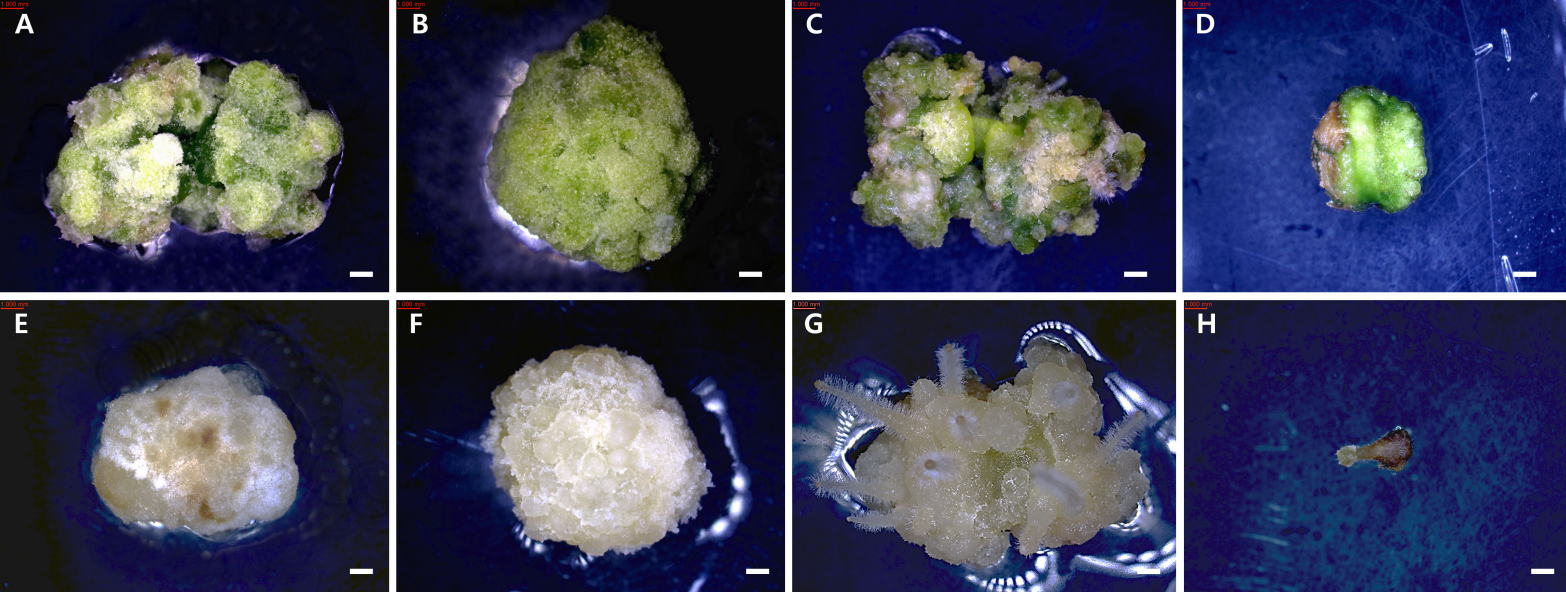
Callus development from *in vitro* plantlet petiole explants of *T. daniellii* after 6 weeks of culture under different media and light conditions. **(A–D)** Cultured under light conditions on MS, WPM, DKW, and SH media, respectively. **(E–H)** Cultured under dark conditions on MS, WPM, DKW, and SH media, respectively. Scale bar represents 1 mm.

The developmental stage of callus varied depending on the culture medium, with generally higher values observed under light conditions than under dark conditions. Callus cultured on WPM medium exhibited the highest developmental stages under both light (4.0) and dark (3.9) conditions. MS and DKW media also showed relatively high developmental stages, ranging from 3.4 to 3.7 (**Figure 5**). In contrast, callus on SH medium showed the lowest stages under both dark (1.4) and light (1.8) conditions.

Redifferentiation rates were significantly affected by medium (F = 26.868, *p* < 0.001), with no significant effects of light condition or their interaction. The DKW medium showed the highest redifferentiation rates at 70.0% (light) and 76.7% (dark), followed by WPM medium at 60.0% (light) and 50.0% (dark), both of which were significantly higher than those observed in MS and SH media (*p* < 0.05). In contrast, redifferentiation rates in MS and SH media were consistently low (≤ 6.7%) under all conditions.

Overall, WPM medium consistently showed high callus induction rates and advanced developmental stages under both light and dark conditions, whereas the highest redifferentiation rates were observed in DKW medium. SH medium exhibited the lowest values for all three parameters among all tested media.

### 3.2 Alkaloid accumulation in callus and tissues

#### 3.2.1 Rutaecarpine content

**Figure 6:**
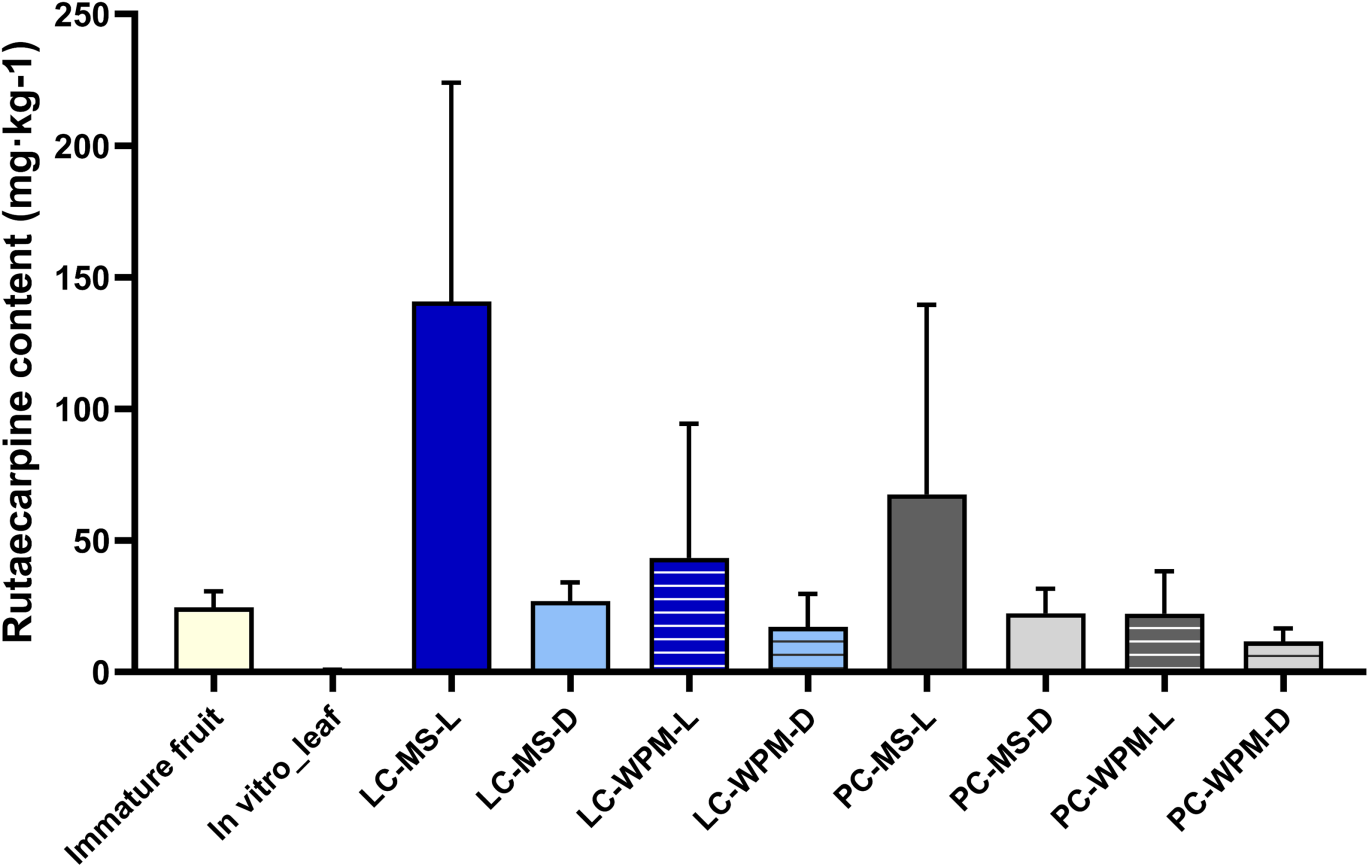
Rutaecarpine content (mg·kg-1) in *in vitro* plantlet leaf tissue and callus derived from *in vitro*-propagated leaf and petiole explants under different culture conditions. Error bars represent standard deviation (n = 3). LC and PC indicate leaf-derived callus and petiole-derived callus, respectively; MS and WPM represent Murashige and Skoog medium and Woody Plant Medium; L and D indicate light and dark culture conditions, respectively.

Analysis of rutaecarpine content revealed that callus induced from leaves of *in vitro*-grown *T*. *daniellii* cultured on MS medium under light conditions exhibited the highest value at 140.83 mg·kg^-1^, significantly higher than all other conditions (**Figure 6**). This level was approximately 5.73 times higher than the rutaecarpine content measured in immature fruit (24.57 mg·kg^-1^) and approximately 216 times higher than that in the leaf tissue of *in vitro*-grown plantlet (0.65 mg·kg^-1^). One-way ANOVA of the 10 treatments indicated an overall difference among treatments (F = 3.226, *p =* 0.014). However, given the large variability and overlapping standard deviation bars, pairwise differences among treatments should be interpreted with caution.

Two-way ANOVA conducted on leaf-derived callus showed that rutaecarpine content was statistically affected by light conditions (F = 6.052, *p =* 0.039): the mean content under light conditions was 92.08 mg·kg^-1^, approximately 4.2 times higher than that under dark conditions (22.10 mg·kg^-1^). By contrast, neither medium composition nor medium x light interaction was statistically significant. Nevertheless, the mean rutaecarpine content in MS medium (83.94 mg·kg^-1^) was approximately 2.8 times higher than that in WPM medium (30.25 mg·kg^-1^).

In petiole-derived callus, two-way ANOVA revealed no statistically significant effects of culture medium (F = 1.706, *p* = 0.228), light condition (F = 1.682, *p =* 0.231), or their interaction (F = 0.655, *p =* 0.442) on rutaecarpine content. Nonetheless, petiole-derived callus cultured on MS medium under light conditions exhibited a relatively high rutaecarpine content of 67.61 mg·kg^-1^, and the mean contents under light conditions (44.89 mg·kg^-1^) was approximately 2.6 times higher than that under dark conditions (17.02 mg·kg^-1^).

#### 3.2.2 Evodiamine content

**Figure 7:**
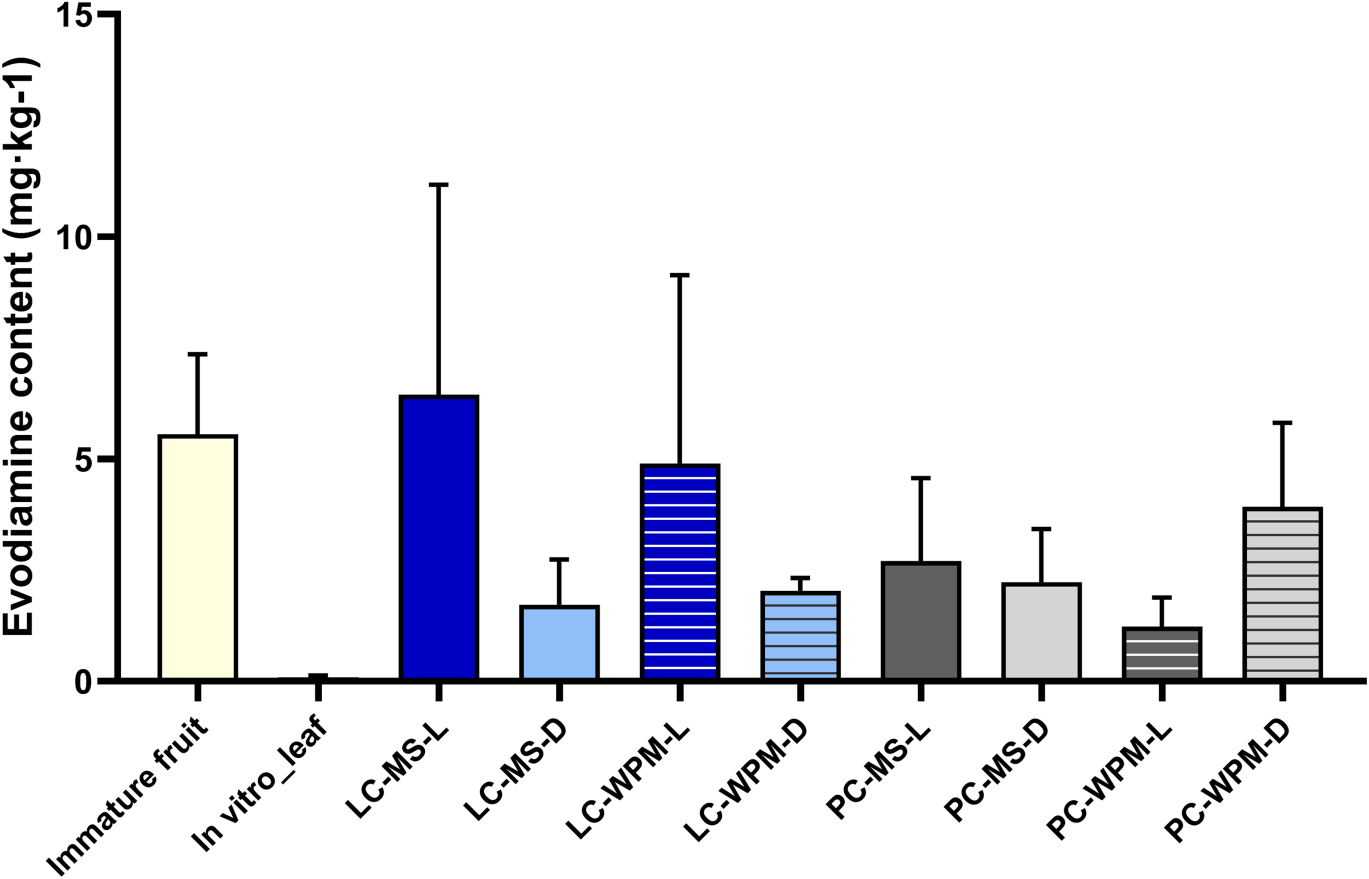
Evodiamine content (mg·kg-1) in *in vitro* plantlet leaf tissue and callus derived from *in vitro*-propagated leaf and petiole explants under different culture conditions. Error bars represent standard deviation (n = 3). No statistically significant differences were observed among treatments based on one-way ANOVA (*p* = 0.054). LC and PC indicate leaf-derived callus and petiole-derived callus, respectively; MS and WPM represent Murashige and Skoog medium and Woody Plant Medium; L and D indicate light and dark culture conditions, respectively.

Callus induced from leaves of *in vitro*-grown plantlet cultured on MS medium under light conditions exhibited the highest evodiamine content at 6.45 mg·kg^-1^ among all conditions, approximately 1.16 times higher than the evodiamine content in immature fruit (5.56 mg·kg^-1^). In contrast, samples directly extracted from leaf tissue of *in vitro*-grown plantlet showed a very low evodiamine content of 0.09 mg·kg^-1^ (**Figure 7**). One-way ANOVA indicated that the difference in evodiamine content among treatments was not statistically significant (F = 2.349, *p =* 0.054), although a marginal trend was observed.

Two-way ANOVA on leaf-derived callus showed that evodiamine content was not significantly affected by medium composition, light conditions, or their interaction. Nevertheless, the mean evodiamine content under light conditions (5.67 mg·kg^-1^) was approximately three times higher than that under dark conditions (1.88 mg·kg^-1^), and leaf-derived callus cultured on WPM medium under light conditions showed an accumulation of 4.90 mg·kg^-1^, comparable to that observed in immature fruit samples.

Similarly, petiole-derived callus showed no significant differences in evodiamine content based on medium composition, light conditions, or their interaction. Among petiole-derived callus, the highest evodiamine content was observed under WPM medium under dark conditions (3.92 mg·kg^-1^), followed by MS medium light conditions (2.71 mg·kg^-1^), MS medium dark conditions (2.23 mg·kg^-1^), and WPM medium light conditions (1.23 mg·kg^-1^) in descending order.

## 4 Discussion

In this study, WPM medium consistently showed high induction rates and stable developmental stages in both leaf and petiole explants in *Tetradium daniellii* regardless of light conditions. This result suggests that culture medium composition may exert a stronger influence on morphogenic responses than light under the tested conditions. Furthermore, the relatively high redifferentiation rates observed in leaf tissues on WPM and in petiole tissue on DKW medium suggest that the combination of 2,4-D and BAP as plant growth regulators (PGRs) promoted organogenic responses.

During callus formation, PGRs act as key factors not only in cell proliferation but also in maintaining the dedifferentiated state and determining the direction of cell differentiation. When the balance of PGRs is suboptimal, callus may fail to maintain a stable dedifferentiated state and instead shift toward redifferentiation (Zavala-Ortiz et al., 2024). The high redifferentiation rates observed in specific media may indicate that the applied PGR conditions, in combination with nutrient composition, favored organogenic pathways rather than the establishment of fully dedifferentiated callus cultures. Such responses may be determined by the complex interaction between the nutritional composition of the medium and the PGR combination (Mishra et al., 2018). In addition, light conditions may also influence cell differentiation and metabolic regulation.

Callus proliferation and alkaloid accumulation were not directly proportional. In plant cell and organ cultures, growth and secondary metabolite biosynthesis are often described as a two-step process (Murthy et al., 2014). Also, callus formation does not necessarily imply the maintenance of specialized metabolic pathways, which are typically associated with differentiated tissues.

Evodiamine and rutaecarpine have been widely reported to accumulate predominantly in fruits of *Evodia rutaecarpa* (Kamikado et al., 1978; Yang et al., 2006; Xiao et al., 2023). Consistent with these reports, immature fruits of *T*. *daniellii* exhibited the highest alkaloid levels among differentiated tissues, with evodiamine and rutaecarpine reaching 5.56 mg/kg and 24.57 mg/kg respectively. In contrast, lower concentrations were observed in vegetative tissues like leaves of *in vitro*-cultured plants.

Alkaloid accumulation in fruits can be explained from both ecological and physiological perspectives. Plants preferentially allocate chemical defenses to tissues where herbivore damage would have the greatest impact on fitness (McKey, 1974). In addition, phytochemical studies on *Evodia rutaecarpa* have demonstrated that fruits contain a diverse array of alkaloids, reinforcing their role as major sites of secondary metabolite accumulation (Li et al., 2020). In many plant species, alkaloid biosynthesis is spatially restricted to specific differentiated cell types and often requires coordinated cellular organization. *In vitro* callus cultures are generally considered limited in their ability to faithfully replicate *in vivo* biosynthetic pathways (Zavala‐Ortiz et al., 2025). However, in the present study, callus culture derived from *in vitro*-grown leaf tissues exhibited substantial accumulation of evodiamine and rutaecarpine, exceeding the levels observed in the original leaf tissues. This observation may suggest either partial retention of biosynthetic capacity from the source tissues or the activation of alternative metabolic pathways during the dedifferentiation process.

Nevertheless, the possibility of residual differentiated tissues or partially organized structures contributed to the observed alkaloid accumulation cannot be excluded. Therefore, the observed alkaloid accumulation in callus should be interpreted with caution and does not necessarily confirm active biosynthesis within fully dedifferentiated cells. Further studies, including repeated subculturing, histological validation, and metabolic tracing approaches are required to confirm the origin of alkaloid production.

The higher accumulation of alkaloids observed in MS medium may be related to its distinct nutrient composition, including nitrogen availability. Differences in nutrient composition can influence ion balance and nutrient uptake efficiency, thereby affecting metabolic flux toward secondary metabolite production (Ferreira and Costa, 2006; Volkov, 2015; Richter et al., 2018). In addition, the relatively high salt concentration of MS medium may impose mild abiotic stress, which has been reported to stimulate secondary metabolites accumulation (Kumar and Sharma, 2018; Jan et al., 2021; Assaf et al., 2022). These results suggest that the enhanced metabolite production observed under MS conditions is likely associated with stress-induced metabolic regulation. However, additional experiments separating the effects of nitrogen concentration, nitrogen form, and overall salt composition are necessary to clarify these mechanisms.

Light also played an important role in alkaloid accumulation. Rutaecarpine content increased significantly under light conditions, whereas evodiamine showed a moderate, but non-significant increase. Light not only provides energy for growth but may also act as an abiotic elicitor, enhancing secondary metabolite accumulation *in vitro* (Zhang et al., 2021). This effect may be associated with the activation of light-responsive signaling pathways that regulate secondary metabolism. In addition, light exposure in the culture conditions may induce oxidative signaling and stress-related responses that are associated with enhanced production of defense compounds (Isah, 2019; Wu et al., 2025). However, the direct regulatory mechanisms linking light to alkaloid biosynthesis in *T. daniellii* remain unclear, and the observed effects are likely indirect. These findings suggest that light promotes secondary metabolite accumulation under *in vitro* conditions.

Taken together, this study shows that callus induction conditions influence both growth and alkaloid accumulation in *T*. *daniellii*. Callus derived from leaf tissues cultured on MS medium under light conditions showed the highest mean accumulation of rutaecarpine and evodiamine among the tested conditions. Importantly, these findings should not be interpreted as definitive evidence of *de novo* alkaloid biosynthesis in callus cultures. Future studies should focus on establishing stable and homogenous callus lines through repeated subculturing, as well as validating metabolite biosynthesis using histological analysis and metabolic tracing techniques. Such approaches will be essential to evaluate the potential of callus cultures as a controlled system for alkaloid accumulation in *T. daniellii*.

## 5 Conclusion

This study identified key culture conditions influencing callus induction and alkaloid accumulation in *Tetradium daniellii*, representing the first systematic evaluation of these processes in this species. The results demonstrate that callus morphogenic responses are highly dependent on culture medium composition. WPM medium showed 100% callus induction in both leaf and petiole explants, whereas SH was unsuitable. In contrast, DKW medium promoted organogenic differentiation, including root formation, indicating a pronounced tendency toward redifferentiation. These findings reveal that *T. daniellii* is highly sensitive to culture medium composition and inherently prone to redifferentiation, which may limit the maintenance of a fully dedifferentiated callus state under standard conditions. Such characteristics should be considered critical constraints when developing *in vitro* production systems based on callus culture.

In terms of secondary metabolite production, callus derived from *in vitro* leaf tissues cultured on MS medium under light conditions exhibited the highest accumulation of rutaecarpine and evodiamine. In most cases, higher alkaloid levels were observed under light conditions, indicating that light plays a key regulatory role in alkaloid accumulation in this species. Based on these results, we propose that alkaloid accumulation is not exclusively associated with complete dedifferentiation but is more likely linked to partially differentiated cellular states or to metabolic reprogramming induced by specific culture conditions, particularly light and nutrient composition. Collectively, this study highlights both the potential and limitations of callus culture as a controllable platform for alkaloid production in *T. daniellii.* To develop a more reliable *in vitro* production system, future studies should focus on optimizing plant growth regulator combinations to reduce redifferentiation and maintain stable callus, establishing suspension culture systems for scalable and uniform biomass production, and performing molecular analysis, such as gene expression profiling, to better understand the regulation of alkaloid biosynthesis.

## Data Availability

The original contributions presented in the study are included in the article/**Supplementary Material**. Further inquiries can be directed to the corresponding author.

## Supplementary material

Supplementary Figure 1 Representative LC-MS/MS chromatograms of rutaecarpine and evodiamine. **(A)** Rutaecarpine standard solution (50 ng·mL^-1^), **(B)** Evodiamine standard solution (50 ng·mL^-1^), **(C)** Rutaecarpine in LC-MS-L sample, **(D)** Evodiamine in LC-MS-L sample.
