# Supplementary figures and images for: Optimization of callus culture for enhanced rutaecarpine and evodiamine accumulation in Tetradium daniellii
Source: Front Plant Sci. 2026 May 13;17:1827737. doi: 10.3389/fpls.2026.1827737 (PMC13212274; doi:10.3389/fpls.2026.1827737)

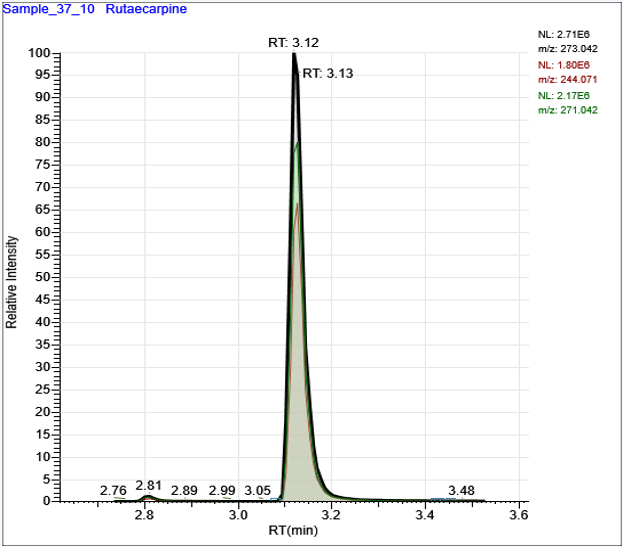

Supplement: Supplementary Figure 1 — Representative LC-MS/MS chromatograms of rutaecarpine and evodiamine. (A) Rutaecarpine standard solution (50 ng·mL-1), (B) Evodiamine standard solution (50 ng·mL-1), (C) Rutaecarpine in LC-MS-L sample, (D) Evodiamine in LC-MS-L sample. [file Image1.tif]

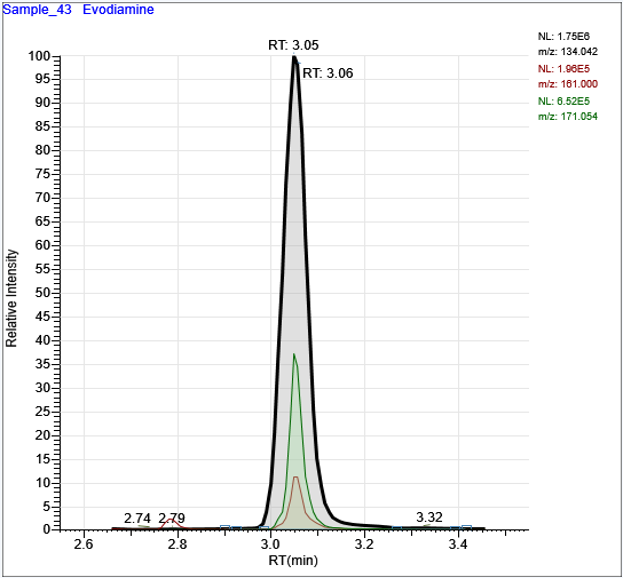

Supplement: Supplementary file 2 [file Image2.tif]
