# Supplementary material for: Optimization of callus culture for enhanced rutaecarpine and evodiamine accumulation in Tetradium daniellii
Source: Front Plant Sci. 2026 May 13;17:1827737. doi: 10.3389/fpls.2026.1827737 (PMC13212274; doi:10.3389/fpls.2026.1827737)
Supplement: Supplementary file 3 [file DataSheet1.zip › Supplementary materials_UHPLC-MSMS/Immature fruit – Rep 1.pdf]

Sample Report

Data File: Immature fruit – Rep 1
Sample ID: 64
Diln Factor: 1.00
Comments:

Tune Report Date: Tune report not found
Instrument ID: Thermo Scientific Instrument
Vial Number: R:D6

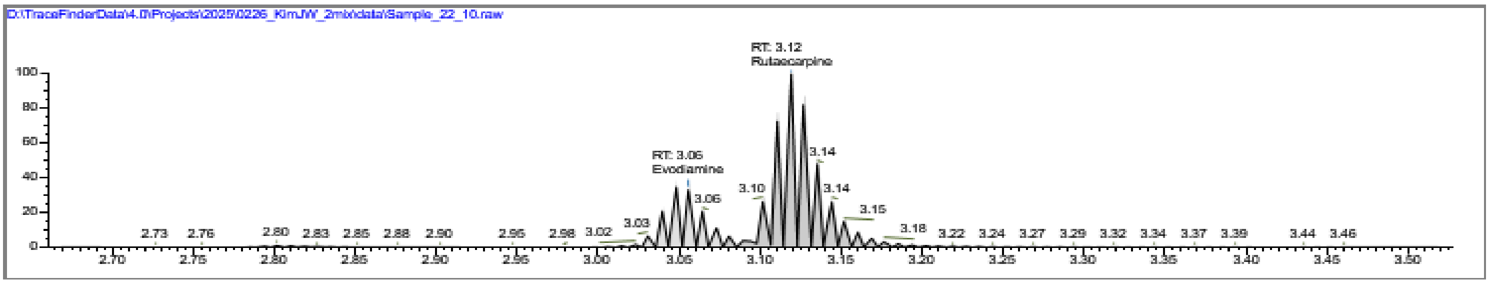

m/z 134.042

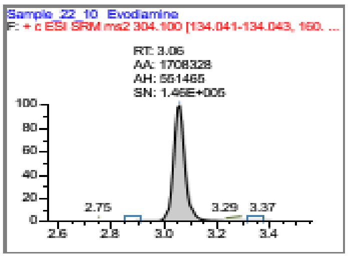

m/z 161.000

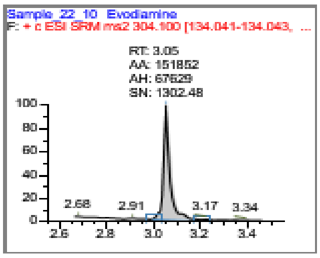

m/z 171.054

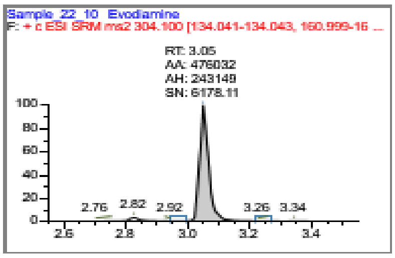

Composite:

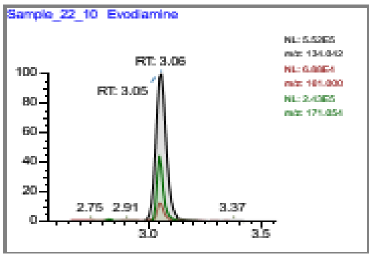

Evodiamine

RT (min)	Ion	Response	Amount	Target Range	Ratio	
			N/A			
3.06	m/z 134.042	1708328	116.639		N/A	I
3.05	m/z 161.000	151852		0.00 - 0.00	8.89 *	
3.05	m/z 171.054	476032		0.00 - 0.00	27.87 *	

m/z 273.042

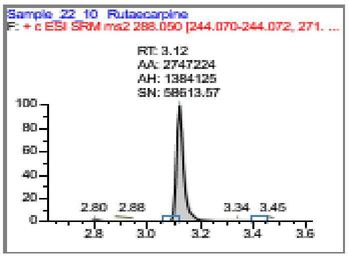

m/z 244.071

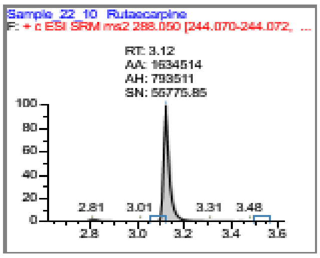

m/z 271.042

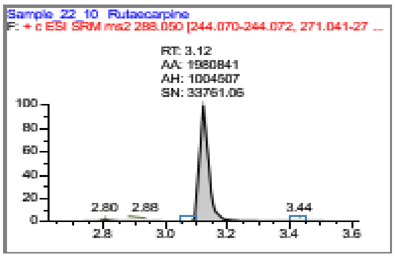

Composite:

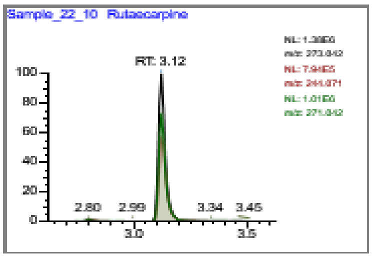

Rutaecarpine

RT (min)	Ion	Response	Amount	Target Range	Ratio	
			N/A			
3.12	m/z 273.042	2747224	435.057		N/A	I
3.12	m/z 244.071	1634514		0.00 - 0.00	59.5 *	
3.12	m/z 271.042	1980841		0.00 - 0.00	72.1 *	
