# Supplementary material for: Optimization of callus culture for enhanced rutaecarpine and evodiamine accumulation in Tetradium daniellii
Source: Front Plant Sci. 2026 May 13;17:1827737. doi: 10.3389/fpls.2026.1827737 (PMC13212274; doi:10.3389/fpls.2026.1827737)
Supplement: Supplementary file 3 [file DataSheet1.zip › Supplementary materials_UHPLC-MSMS/Immature fruit – Rep 3.pdf]

Sample Report

Data File:	Immature fruit – Rep 3	Tune Report Date:	Tune report not found
Cali File:	0226_KimJW_2mix.calx	Operator ID:	Altis
Sample ID:	66	Instrument ID:	Thermo Scientific Instrument
Diln Factor:	1.00	Vial Number:	R:D8
Comments:			

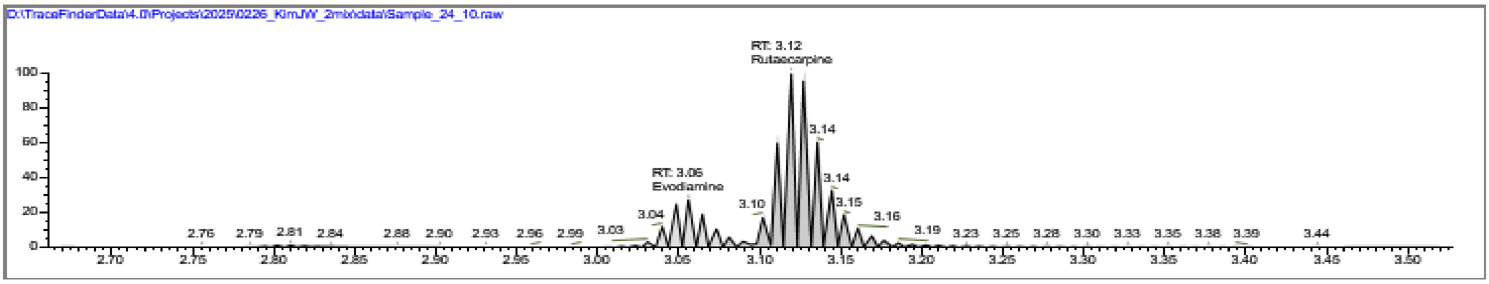

m/z 134.042 **m/z 161.000** **m/z 171.054** **Composite:**

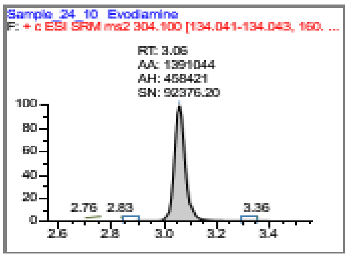

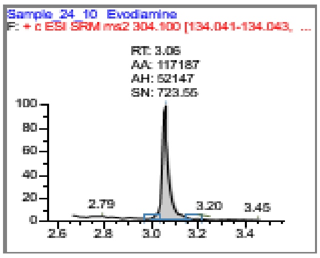

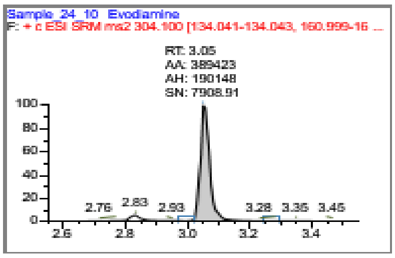

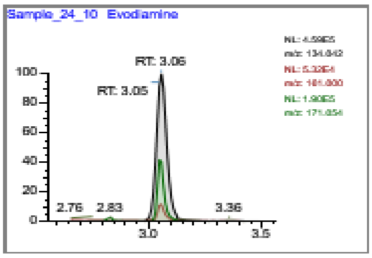

Evodiamine

RT (min)	Ion	Response	Amount	Target Range	Ratio	
			N/A			
3.06	m/z 134.042	1391044	94.790		N/A	I
3.06	m/z 161.000	117187		0.00 - 0.00	8.42 *	
3.05	m/z 171.054	389423		0.00 - 0.00	28 *	

m/z 273.042 **m/z 244.071** **m/z 271.042** **Composite:**

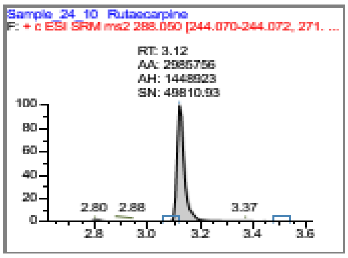

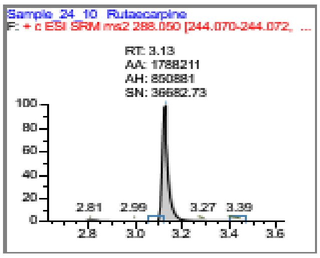

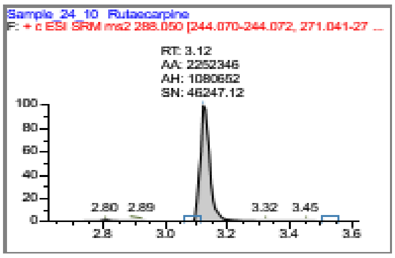

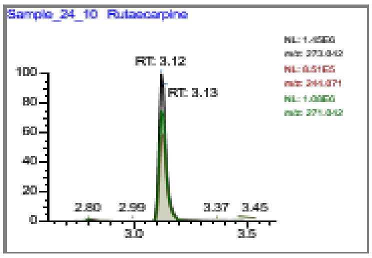

Rutaecarpine

RT (min)	Ion	Response	Amount	Target Range	Ratio	
			N/A			
3.12	m/z 273.042	2985756	472.749		N/A	I
3.13	m/z 244.071	1788211		0.00 - 0.00	59.89 *	
3.12	m/z 271.042	2252346		0.00 - 0.00	75.44 *	
