# Supplementary material for: Optimization of callus culture for enhanced rutaecarpine and evodiamine accumulation in Tetradium daniellii
Source: Front Plant Sci. 2026 May 13;17:1827737. doi: 10.3389/fpls.2026.1827737 (PMC13212274; doi:10.3389/fpls.2026.1827737)
Supplement: Supplementary file 3 [file DataSheet1.zip › Supplementary materials_UHPLC-MSMS/In vitro_leaf– Rep 2.pdf]

Sample Report

Data File:	In vitro_leaf– Rep 2	Tune Report Date:	Tune report not found
Cali File:	0416_KimJW_2mix.calx	Operator ID:	Altis
Sample ID:	43	Instrument ID:	Thermo Scientific Instrument
Diln Factor:	1.00	Vial Number:	R:D1
Comments:			

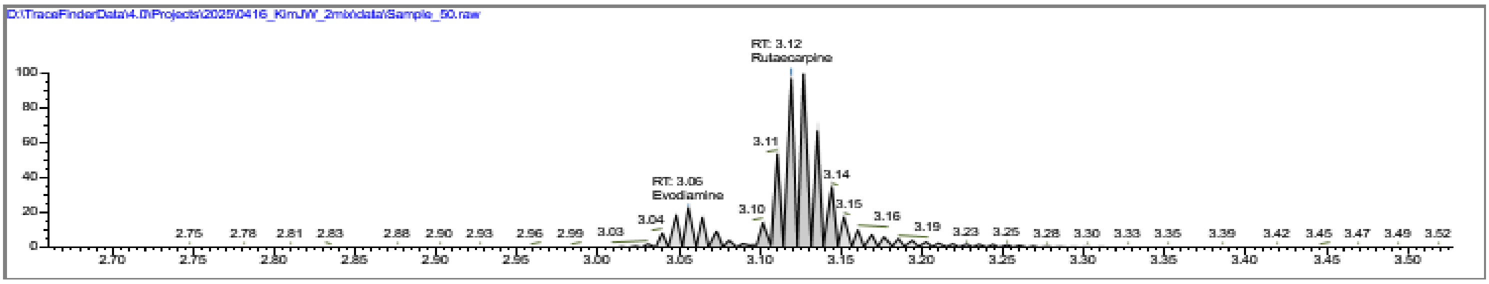

m/z 134.042 | **m/z 161.000** | **m/z 171.054** | **Composite:**

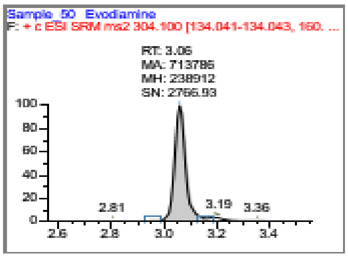

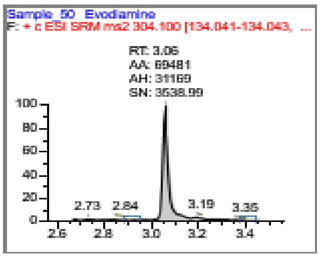

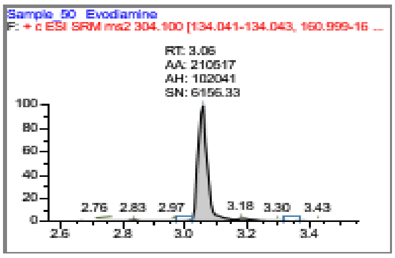

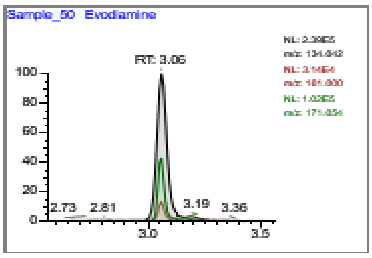

Evodiamine

RT (min)	Ion	Response	Amount	Target Range	Ratio	
			N/A			
3.06	m/z 134.042	713786 M	23.385		95.86	I
3.06	m/z 161.000	69481		0.00 - 0.00	9.73 *	
3.06	m/z 171.054	210517		0.00 - 0.00	29.49 *	

m/z 273.042 | **m/z 244.071** | **m/z 271.042** | **Composite:**

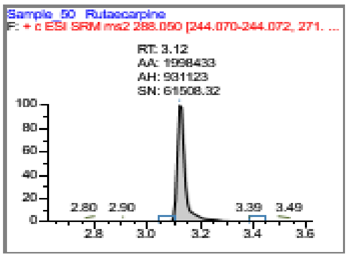

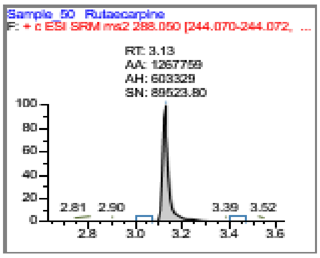

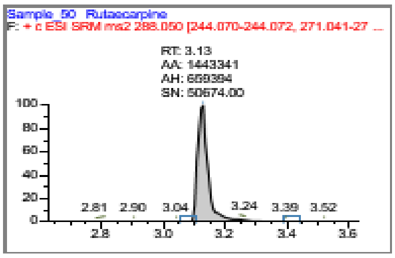

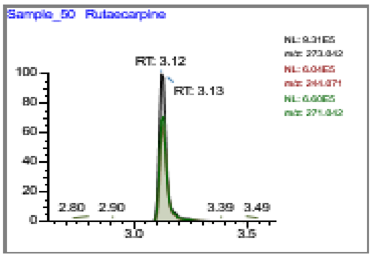

Rutaecarpine

RT (min)	Ion	Response	Amount	Target Range	Ratio	
			N/A			
3.12	m/z 273.042	1998433	129.565		N/A	I
3.13	m/z 244.071	1267759		0.00 - 0.00	63.44 *	
3.13	m/z 271.042	1443341		0.00 - 0.00	72.22 *	
