# Supplementary material for: Optimization of callus culture for enhanced rutaecarpine and evodiamine accumulation in Tetradium daniellii
Source: Front Plant Sci. 2026 May 13;17:1827737. doi: 10.3389/fpls.2026.1827737 (PMC13212274; doi:10.3389/fpls.2026.1827737)
Supplement: Supplementary file 3 [file DataSheet1.zip › Supplementary materials_UHPLC-MSMS/LC-MS-D – Rep 1 - Rutaecarpine.pdf]

Sample Report

Data File:	LC-MS-D – Rep 1 - Rutaecarpine	Tune Report Date:	Tune report not found
Cali File:	0226_KimJW_2mix.calx	Operator ID:	Altis
Sample ID:	86	Instrument ID:	Thermo Scientific Instrument
Diln Factor:	1.00	Vial Number:	R:F9
Comments:			

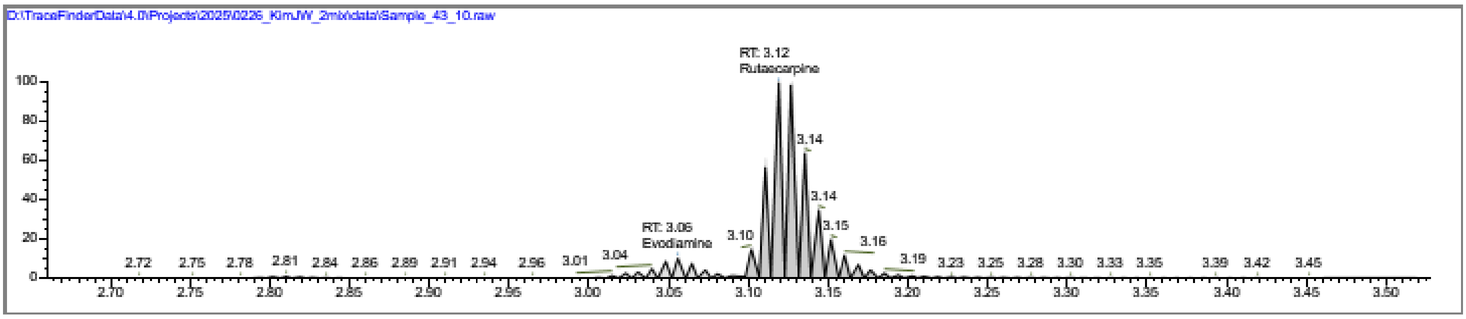

m/z 273.042

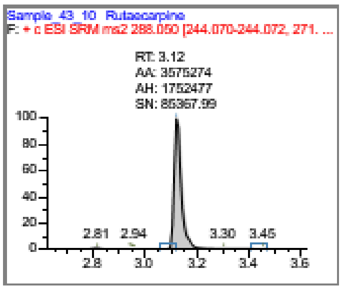

m/z 244.071

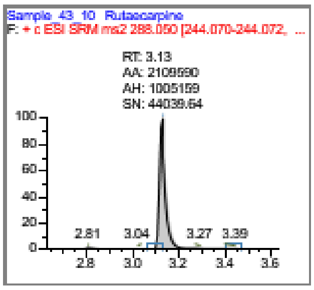

m/z 271.042

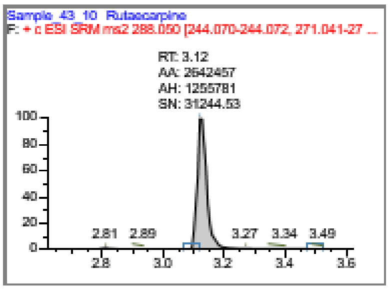

Composite:

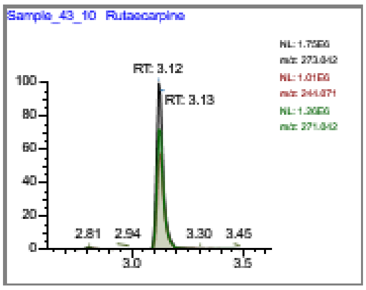

Rutaecarpine

RT (min)	Ion	Response	Amount	Target Range	Ratio	
			N/A			
3.12	m/z 273.042	3575274	565.903		N/A	I
3.13	m/z 244.071	2109590		0.00 - 0.00	59.01 *	
3.12	m/z 271.042	2642457		0.00 - 0.00	73.91 *	
