# Supplementary material for: Optimization of callus culture for enhanced rutaecarpine and evodiamine accumulation in Tetradium daniellii
Source: Front Plant Sci. 2026 May 13;17:1827737. doi: 10.3389/fpls.2026.1827737 (PMC13212274; doi:10.3389/fpls.2026.1827737)
Supplement: Supplementary file 3 [file DataSheet1.zip › Supplementary materials_UHPLC-MSMS/LC-MS-L – Rep 3 - Evodiamine.pdf]

Sample Report

Data File:	LC-MS-L – Rep 3 - Evodiamine	Tune Report Date:	Tune report not found
Cali File:	0226_KimJW_2mix.calx	Operator ID:	Altis
Sample ID:	82	Instrument ID:	Thermo Scientific Instrument
Diln Factor:	1.00	Vial Number:	R:F5
Comments:			

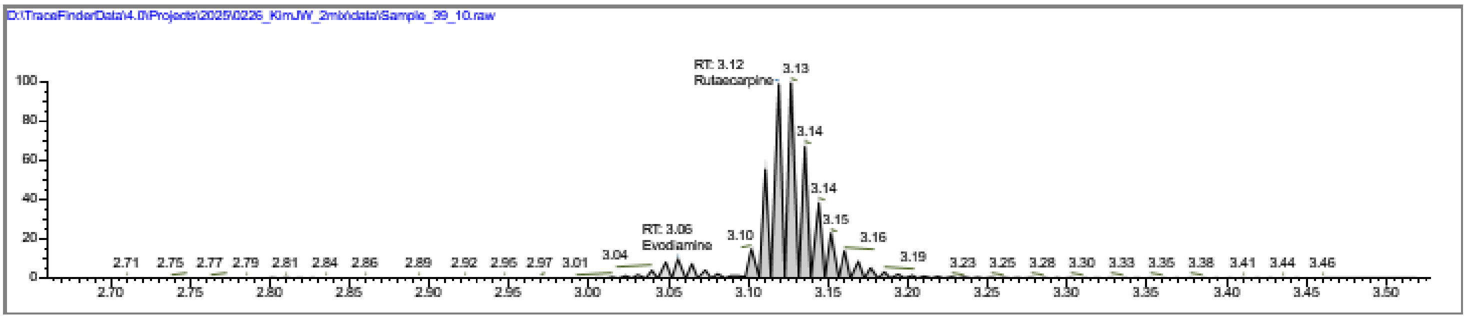

m/z 134.042

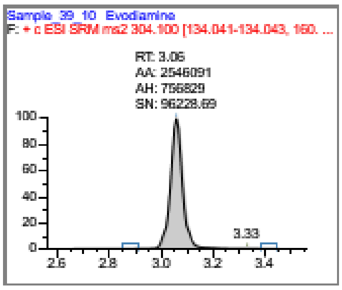

m/z 161.000

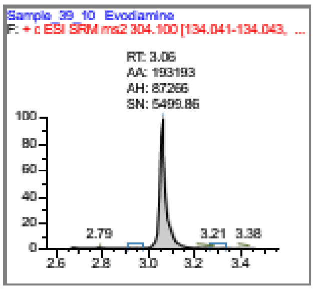

m/z 171.054

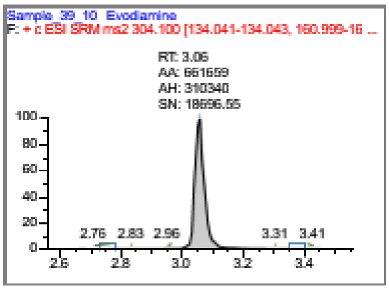

Composite:

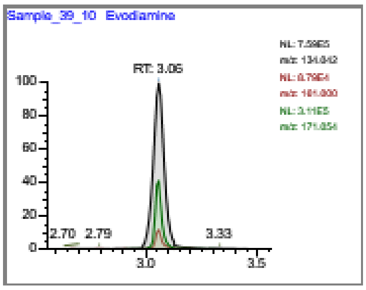

Evodiamine

RT (min)	Ion	Response	Amount	Target Range	Ratio	
			N/A			
3.06	m/z 134.042	2546091	174.329		N/A	I
3.06	m/z 161.000	193193		0.00 - 0.00	7.59 *	
3.06	m/z 171.054	661659		0.00 - 0.00	25.99 *	
