# Supplementary material for: Optimization of callus culture for enhanced rutaecarpine and evodiamine accumulation in Tetradium daniellii
Source: Front Plant Sci. 2026 May 13;17:1827737. doi: 10.3389/fpls.2026.1827737 (PMC13212274; doi:10.3389/fpls.2026.1827737)
Supplement: Supplementary file 3 [file DataSheet1.zip › Supplementary materials_UHPLC-MSMS/PC-MS-L – Rep 3- Evodiamine.pdf]

Sample Report

Data File:	PC-MS-L – Rep 3- Evodiamine	Tune Report Date:	Tune report not found
Cali File:	0226_KimJW_2mix.calx	Operator ID:	Altis
Sample ID:	184	Instrument ID:	Thermo Scientific Instrument
Diln Factor:	1.00	Vial Number:	G:D5
Comments:			

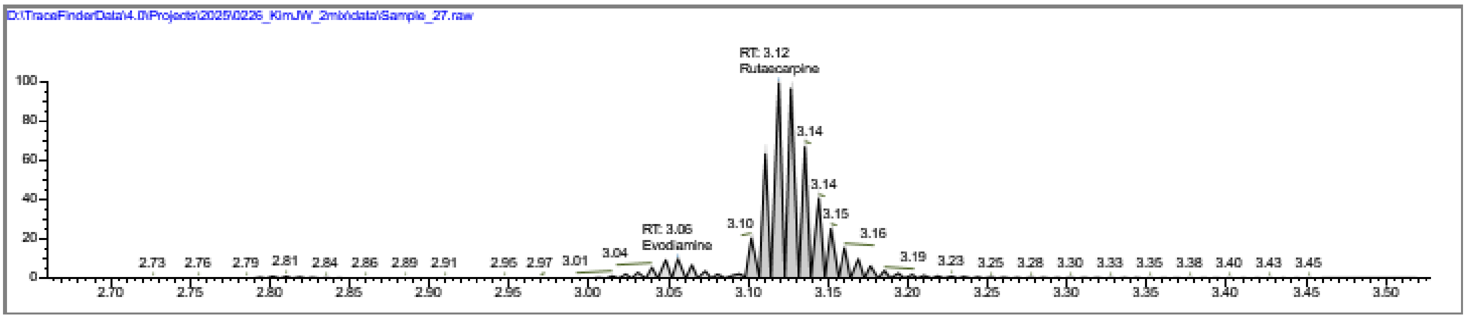

m/z 134.042

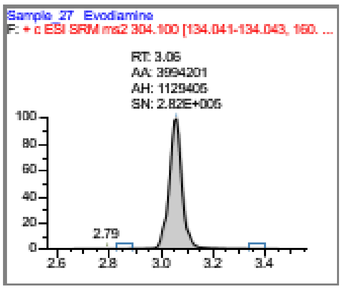

m/z 161.000

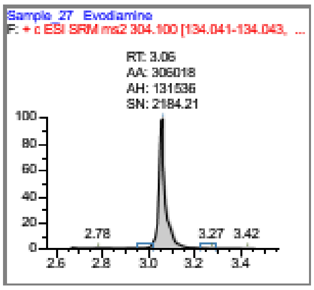

m/z 171.054

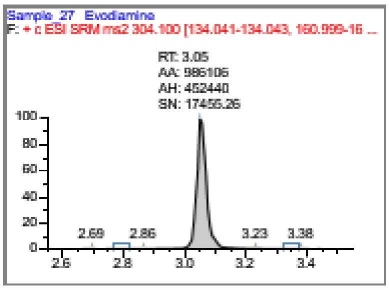

Composite:

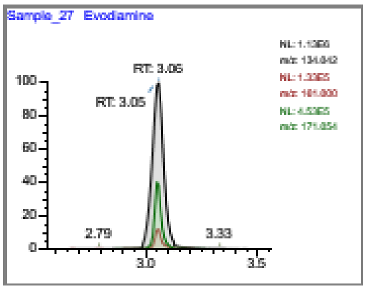

Evodiamine

RT (min)	Ion	Response	Amount	Target Range	Ratio	
			N/A			
3.06	m/z 134.042	3994201	274.049		N/A	I
3.06	m/z 161.000	306018		0.00 - 0.00	7.66 *	
3.05	m/z 171.054	986106		0.00 - 0.00	24.69 *	
