# Supplementary material for: Optimization of callus culture for enhanced rutaecarpine and evodiamine accumulation in Tetradium daniellii
Source: Front Plant Sci. 2026 May 13;17:1827737. doi: 10.3389/fpls.2026.1827737 (PMC13212274; doi:10.3389/fpls.2026.1827737)
Supplement: Supplementary file 3 [file DataSheet1.zip › Supplementary materials_UHPLC-MSMS/PC-MS-D – Rep 2- Rutaecarpine.pdf]

# Sample Report

| | | | |
|---|---|---|---|
| Data File: | PC-MS-D – Rep 2- Rutaecarpine | Tune Report Date: | Tune report not found |
| Cali File: | 0226_KimJW_2mix.calx | Operator ID: | Altis |
| Sample ID: | 75 | Instrument ID: | Thermo Scientific Instrument |
| Diln Factor: | 1.00 | Vial Number: | R:E7 |
| Comments: | | | |

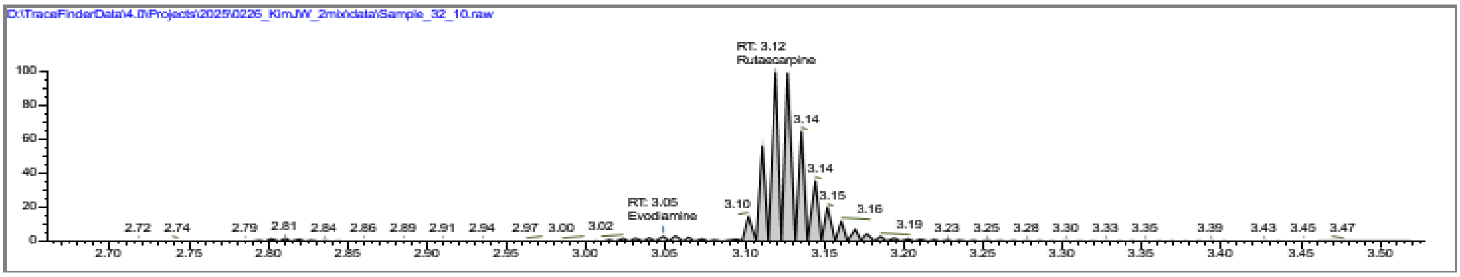


**m/z 273.042**

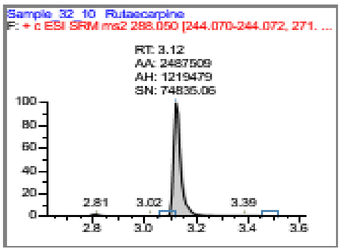


**m/z 244.071**

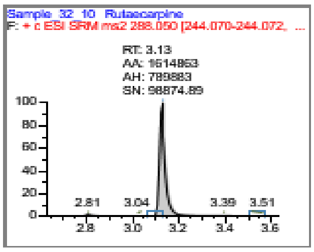


**m/z 271.042**

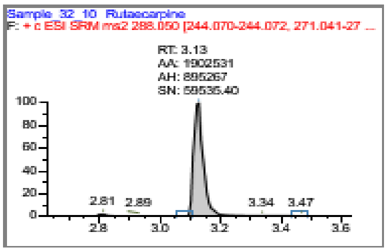


**Composite:**

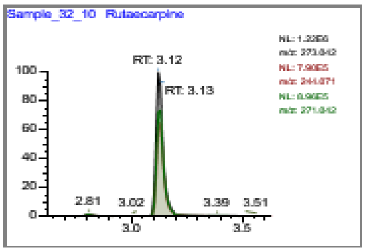


**Rutaecarpine**

| RT (min) | Ion | Response | Amount | Target Range | Ratio | |
|---|---|---|---|---|---|---|
| | | | N/A | | | |
| 3.12 | m/z 273.042 | 2487509 | 394.017 | | N/A | I |
| 3.13 | m/z 244.071 | 1614863 | | 0.00 - 0.00 | 64.92 * | |
| 3.13 | m/z 271.042 | 1902531 | | 0.00 - 0.00 | 76.48 * | |
