# Supplementary material for: Optimization of callus culture for enhanced rutaecarpine and evodiamine accumulation in Tetradium daniellii
Source: Front Plant Sci. 2026 May 13;17:1827737. doi: 10.3389/fpls.2026.1827737 (PMC13212274; doi:10.3389/fpls.2026.1827737)
Supplement: Supplementary file 3 [file DataSheet1.zip › Supplementary materials_UHPLC-MSMS/LC-WPM-D – Rep 1-Evodiamine.pdf]

Sample Report

Data File:	LC-WPM-D – Rep 1-Evodiamine	Tune Report Date:	Tune report not found
Cali File:	0226_KimJW_2mix.calx	Operator ID:	Altis
Sample ID:	184	Instrument ID:	Thermo Scientific Instrument
Diln Factor:	1.00	Vial Number:	G:F6
Comments:			

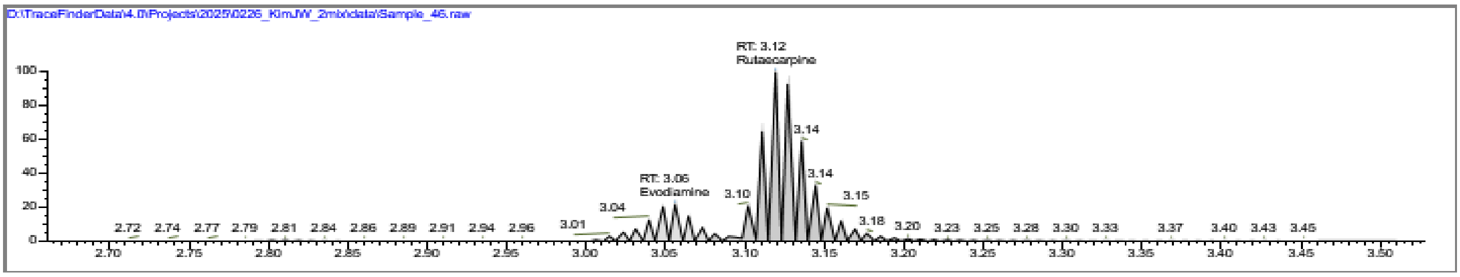

m/z 134.042

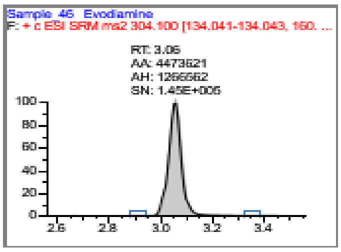

m/z 161.000

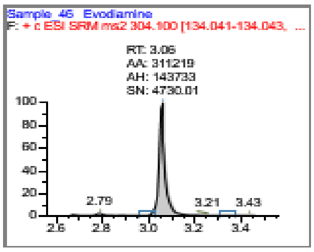

m/z 171.054

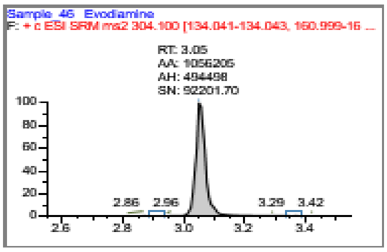

Composite:

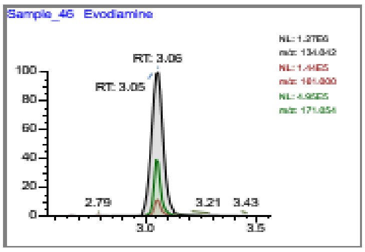

Evodiamine

RT (min)	Ion	Response	Amount	Target Range	Ratio	
			N/A			
3.06	m/z 134.042	4473621	307.063		N/A	I
3.06	m/z 161.000	311219		0.00 - 0.00	6.96 *	
3.05	m/z 171.054	1056205		0.00 - 0.00	23.61 *	
