# Supplementary material for: Optimization of callus culture for enhanced rutaecarpine and evodiamine accumulation in Tetradium daniellii
Source: Front Plant Sci. 2026 May 13;17:1827737. doi: 10.3389/fpls.2026.1827737 (PMC13212274; doi:10.3389/fpls.2026.1827737)
Supplement: Supplementary file 3 [file DataSheet1.zip › Supplementary materials_UHPLC-MSMS/PC-WPM-L – Rep 3- Evodiamine.pdf]

Sample Report

Data File: PC-WPM-L – Rep 3- Evodiamine		Tune Report Date:	Tune report not found
Cali File: 0226_KimJW_2mix.calx		Operator ID:	Altis
Sample ID: 184		Instrument ID:	Thermo Scientific Instrument
Diln Factor: 1.00		Vial Number:	G:D8
Comments:			

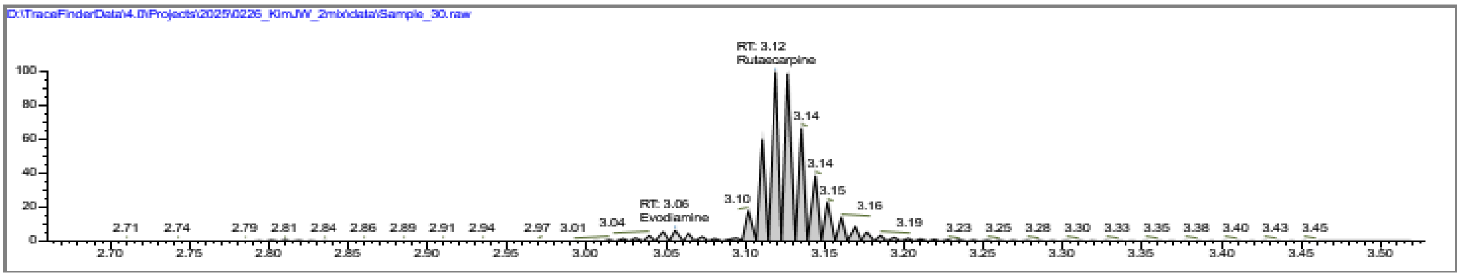

m/z 134.042 | **m/z 161.000** | **m/z 171.054** | **Composite:**

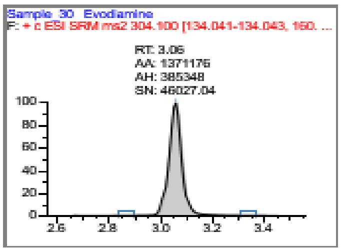

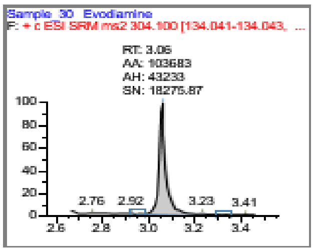

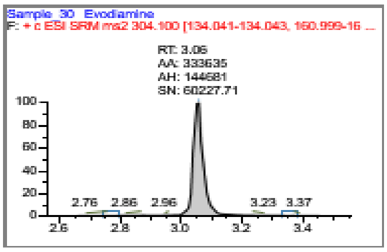

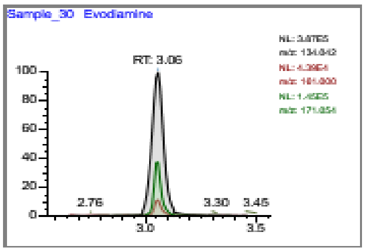

Evodiamine

RT (min)	Ion	Response	Amount	Target Range	Ratio	
			N/A			
3.06	m/z 134.042	1371176	93.422		N/A	I
3.06	m/z 161.000	103683		0.00 - 0.00	7.56 *	
3.06	m/z 171.054	333635		0.00 - 0.00	24.33 *	
