# Supplementary material for: Optimization of callus culture for enhanced rutaecarpine and evodiamine accumulation in Tetradium daniellii
Source: Front Plant Sci. 2026 May 13;17:1827737. doi: 10.3389/fpls.2026.1827737 (PMC13212274; doi:10.3389/fpls.2026.1827737)
Supplement: Supplementary file 4 [file Table1.docx]

**Supplementary materials**

Table S1. Inorganic salt and vitamin composition (mg·L^-1^) of MS, WPM, DKW, and SH media.

| **Composition** | **Concentration (mg·L^-1^)** | | | |
| --- | --- | --- | --- | --- |
|  | **MS** | **WPM** | **DKW** | **SH** |
| **Micro Elements** |  |  |  |  |
| CoCl_2·_6H_2_O | 0.025 | - | - | 0.10 |
| CuSO_4_·5H_2_O | 0.025 | 0.25 | 0.25 | 0.20 |
| FeNaEDTA | 36.70 | 36.70 | 44.63 | 19.80 |
| H_3_BO_3_ | 6.20 | 6.20 | 4.80 | 5.00 |
| KI | 0.83 | - | - | 1.00 |
| MnSO_4_·H_2_O | 16.90 | 22.30 | 33.80 | 10.00 |
| Na_2_MoO_4_·2H_2_O | 0.25 | 0.25 | 0.39 | 0.10 |
| ZnSO_4_·7H_2_O | 8.60 | 8.60 | 17.00 | 1.00 |
| **Macro Elements** |  |  |  |  |
| CaCl_2_ | 332.02 | 72.50 | 112.50 | 151.00 |
| Ca(NO_3_)2·4H_2_O | - | 471.26 | 1664.64 | - |
| KH_2_PO_4_ | 170.00 | 170.00 | 265.00 | - |
| K_2_SO_4_ | - | - | 1559.00 | - |
| KNO_3_ | 1900.00 | 990.00 | - | 2500.00 |
| MgSO_4_ | 180.54 | 180.54 | 361.49 | 195.05 |
| NH_4_NO_3_ | 1650.00 | 400.00 | 1416.00 | - |
| (NH_4_)H_2_PO_4_ | - | - | - | 300.00 |
| **Vitamins** |  |  |  |  |
| Glycine | - | 2.00 | 2.00 | - |
| myo-Inositol | 100.00 | 100.00 | 100.00 | 1000.00 |
| Nicotinic acid | 1.00 | 0.50 | 1.00 | 5.00 |
| Pyridoxine HCl | 1.00 | 0.50 | - | 0.50 |
| Thiamine HCl | 10.00 | 1.00 | 2.00 | 5.00 |
